# Novel Encapsulated Calcium Butyrate Supplement Enhances On-Farm Dairy Calf Growth Performance and Body Conformation in a Pasture-Based Dairy Production System

**DOI:** 10.3390/ani10081380

**Published:** 2020-08-08

**Authors:** Aduli Enoch Othniel Malau-Aduli, Razaq Oladimeji Balogun, John Roger Otto, Sumita Verma, Maduka Wehella, David Jones

**Affiliations:** 1 College of Public Health, Medical and Veterinary Sciences, James Cook University, Townsville, QLD 4811, Australia; john.otto@jcu.edu.au; 2 Kemin Industries (Asia) Pte Limited, 12 Senoko Drive, Singapore 758200, Singapore; razaq.balogun@kemin.com; 3 School of Land and Food, Tasmanian Institute of Agriculture, University of Tasmania, Sandy Bay, Hobart, Tasmania 7001, Australia; sumita.verma@utas.edu.au (S.V.); maduka.wehella@utas.edu.au (M.W.); 4 Willowdene Dairy Farm, Hamilton, Tasmania 7140, Australia; davidjones26@hotmail.com

**Keywords:** heifer calves, encapsulated calcium butyrate, growth performance, body conformation

## Abstract

**Simple Summary:**

The problem addressed in this study was that of enhancing healthy gut development in calves with the aim of promoting early weaning through faster growth using encapsulated calcium butyrate (ECAB, ButiPEARL™, Kemin Industries, Inc., Des Moines, IA, USA). Results showed that supplementation of neonatal calves with ECAB at 4 kg/ton of calf starter feed, under a pasture-based dairy production system, had a significant effect on calf growth and development, presumably through improved rumen and small intestine development, gut health, feed digestibility, and nutrient absorption. This first of its kind on-farm, pasture-based, dairy farmer-driven study makes a significant novel contribution to our understanding of the impact of supplementing neonatal heifer calves with ECAB on pre-weaning and post-weaning growth performance and body conformation traits in purebred Friesian and crossbred Friesian × Swedish Red and Friesian × Jersey. The practical implication is accelerated calf growth and improved lifetime performance.

**Abstract:**

The effect of supplementing neonatal heifer calves with varying levels of ECAB on pre-weaning growth performance was investigated. Post-weaning growth was also measured, to determine any carry-over effect of pre-weaning supplementation of ECAB. Forty-eight heifer calves (7 ± 0.4 days old, average liveweight of 39.3 ± 5.3 kg) were utilized in a complete randomised experimental design, comprising 16 calves per pen, randomly allocated to one of the following three treatments: (1) Basal commercial calf starter mix without ECAB (Control); (2) control plus 4 kg/ton of ECAB (Low); and (3) control plus 6 kg/ton of ECAB (High). Calves were group-fed ad libitum for 77 days (11 weeks, pre-weaning period) with free choice access to water and 5.5 L of milk per head per day through an automated feeder. Calves were weighed weekly during the pre-weaning period, after which all calves were then weaned onto the same ryegrass pasture as one group. At approximately 9 months of age, calves were weighed to estimate post-weaning body weight gain. During the pre-weaning period, average daily dry matter feed intake was similar for 4 kg/ton and 6 kg/ton calves (649 g versus 688 g, respectively) and both were greater than that of the control calves (382 g). Average daily gain (ADG) was significantly higher for 4 kg/ton calves compared to 6 kg/ton calves or control calves (0.83 ± 0.03 kg, 0.74 ± 0.03 kg and 0.71 ± 0.03 kg, respectively; *p* = 0.0001). Similarly, 4 kg/ton calves had significantly increased chest girth (95.9 ± 0.7 cm), withers height (88.9 ± 0.5 cm), body length (82.9 ± 0.6 cm), and body condition score (1.99 ± 0.12) compared to 6 kg/ton calves (93.4 ± 0.7 cm, 87.4 ± 0.7, 81.5 ± 0.6 cm, and 1.67 ± 0.10, respectively) or control calves (92.9 ± 0.7 cm, 88.2 ± 0.5 cm, 80.1 ± 0.6 cm, and 1.30 ± 0.08, respectively). There was significant treatment × week interaction for all pre-weaning growth parameters. Breed differences were detected but there was no treatment × breed interaction. Post-weaning, 4 kg/t calves and 6 kg/t calves had significantly higher ADG compared to control calves (0.80 ± 0.03 kg, 0.85 ± 0.03 kg versus 0.70 ± 0.03 kg, respectively; *p* = 0.0047). It is concluded that under the conditions of this study, supplementing heifer calves with ECAB during pre-weaning period resulted in improved growth performance and there appears to be a post-weaning carry-over effect.

## 1. Introduction

Adequate gut health and development leads to accelerated growth during the rearing stage of a heifer with a considerable long-term effect on its lifetime performance [1]. The early attainment of weaning weight and age at first calving via faster growth and body conformation has major economic implications on heifer rearing costs because a healthy and well-developed heifer is likely to become a profitable and improved lifetime performing cow [2]. It is well established that puberty is triggered by body weight; hence, age at first calving could be affected by the body weight of a heifer calf [3]. Studies have also shown that there is a positive correlation between body weight at calving and milk yield of dairy cows [4], suggesting that there could be a positive relationship between rearing weight or growth rate of heifers and their subsequent milk production [5]. Therefore, a nutritional management program that promotes adequate heifer growth during the rearing stage can have a significantly positive and considerable effect on lifetime performance of dairy heifers.

A nutritional strategy to improve calf gut health and growth performance is supplementation with butyrate. Ruminal butyrate production stimulates rumen epithelial growth, encourages an increase in the rumen mitosis to apoptosis ratio, enables large rumen papillae growth, and increases the surface area for nutrient absorption [6,7,8,9,10,11]. Some studies on supplementing calf starters with salts of butyric acid showed positive effects on the development of the rumen papillae, small intestinal villi, and the overall performance of young calves [12,13,14]. These are consistent with earlier studies which showed that supplementation with butyric acid promoted growth of epithelial cells of the gastro-intestinal tract (GIT), improved digestion of feed, absorption of nutrients, and immune function of the intestines [15]. A well-developed GIT should promote feed intake, nutrient absorption, and overall intestinal health [8], which will in turn enhance growth performance [16]. A study that investigated both milk replacer and calf starter methods of supplementation found that compared to control calves, supplementation through starter feed increased feed intake, whereas supplementation through milk replacer did not [14]. Therefore, in the present study, butyrate was supplemented in the starter feed while fresh milk was allocated at a fixed rate of 5.5 L per head per day through an auto feeder. To the best of our current knowledge of the published literature, there are no existing farmer-driven on-farm studies that have investigated the effect of supplementation with encapsulated calcium butyrate (ECAB) in neonatal calves from pre-weaning to post-weaning, and follow-up carry-over effects to nine months of age in pasture-based dairy production systems in Australia and New Zealand. Therefore, the objective of this novel on-farm study was to investigate the effect of pre-weaning supplementation of neonatal heifer calves with low and high levels of encapsulated calcium butyrate on feed intake, growth performance, and body conformation parameters in a pasture-based dairy system, and to determine any carry-over effects of pre-weaning ECAB supplementation on post-weaning growth.

## 2. Materials and Methods

### 2.1. Experimental Site and Animal Ethics

The use of animals and procedures performed in this study were all approved by the University of Tasmania Animal Ethics Committee (permit no. A15999) and all experiments were performed in accordance with relevant guidelines and regulations of the Animal Ethics Committee. The experiment was conducted at a commercial dairy farm in Hamilton, Tasmania, Australia. Tasmania is characterized by a cool temperate climate and the long-term average minimum and maximum temperatures for Hamilton during the period of this study (September–November) were 4.0–7.3 and 15.7–21.1 °C, respectively. The mean average rainfall and humidity were 71.4 mm and 40%, respectively.

### 2.2. Animals, Treatments, and Experimental Design

Forty-eight (48), purebred Friesian (FR) and crossbreds (Friesian × Jersey, FJ and Friesian × Swedish-Red, FSR) dairy heifer calves (average age of 7 ± 0.4 days), with an initial body weight (BWT) of 39.3 ± 5.3 kg, were used in a randomized complete block design. Sixteen dairy heifer calves were randomly allocated to each of three dietary treatments of commercial calf starter feed (mash) containing encapsulated calcium butyrate (ButiPEARL™, Kemin Industries, Des Moines, IA, USA) at 0 kg/ton (Control), 4 kg/ton (Low ECAB), or 6 kg/ton (High ECAB). The ECAB used in this study is a slow release product, manufactured using a MicroPEARL™ encapsulated technology. It contains a minimum of 45% calcium butyrate, encapsulated in a lipid matrix, allowing for up to 95% release throughout the upper and lower GIT, hence it is expected to have both ruminal and small intestinal effect. The level of supplementation is so small that any appreciable energy contribution is unlikely, rather the effect of butyrate has been suggested to be mainly through DNA signaling and not as an energy source [10]. The inclusion range of ECAB into calf starter rations is 4–8 kg/ton, with each neonatal calf expected to ingest between 600 and 650 g per day of the starter feed. However, based on our preliminary unpublished data, the 8 kg/ton level was excluded due to its negative effect on palatability and intake of the starter feed. Calves were housed as a group in separate pens and had ad libitum access to the starter feed and clean water. Whole milk was offered through an automatic milk feeder at 5.5 L allocation per calf per day. The intake of milk was regulated to allow an even spread over a 24 h period. Each group pen was considered an experimental treatment and individual animals in each pen as replicates because there were repeated measures per animal over the duration of experimental period. Each pen was balanced by dam parity and calf breed.

Calves were fed experimental diet ad libitum for 11 weeks (77 days, pre-weaning period), after which they were weaned, as one group, onto the same ryegrass pasture and supplemented with the control starter feed for up to 6 months of age, and thereafter, solely on pasture. They were weighed at approximately 9 months (corresponding to 36 weeks or 252 days) of age to determine any carry-over effect of pre-weaning supplementation of ECAB on post-weaning growth.

### 2.3. Feed Intake, Body Weight, and Body Conformation Measurements

The amount of starter feed offered and the residual were recorded on a weekly basis and total group starter feed intake was calculated. Average starter feed intake per calf was calculated as the total feed allocated to the group minus the residual feed divided by the number of calves per treatment. During the pre-weaning period, calves were weighed individually on a weekly basis, using an electronic walk-over weighing scale (Sunbeam TRU-Test AG 500 Series, Model 703, Tru-Test Inc, Texas). Weekly BWT was recorded and average daily gain (ADG) calculated. Chest girth (CG), withers height (WH), body length (BL), and BCS were measured on a weekly basis using a standard measuring tape by the same assessor to ensure consistency, repeatability, and accuracy. Body condition scoring was on a standard scale of one to five as per Wildman et al. [17]. Post-weaning body weight of individual calves was measured once, at approximately nine months of age and post-weaning total weight gain and ADG were calculated.

### 2.4. Experimental Diet and Nutrient Analysis

Feed and pasture samples were collected weekly, bulked and thoroughly mixed at the end of the study to provide representative samples for laboratory analysis. Feed samples were sent to Cumberland Valley Analytical Services (Bendigo, Victoria, Australia) to determine the dry matter (DM), neutral detergent fibre (NDF), acid detergent fibre (ADF), crude protein (CP), metabolisable energy (ME), and ether extract (EE) based on AOAC international standards (AOAC 1999. Official Methods of Analysis. AOAC International, Gaithersburg, MD, USA).

### 2.5. Statistical Analysis

Statistical analysis of all collected data was performed in SAS version 9.2 (SAS Institute, Cary, NC, USA). Means, standard deviations, standard errors, minimum, and maximum values were computed using PROC MEANS and scrutinized for any data entry errors. The dependent variables (feed intake, liveweight, average daily gain, and body conformation parameters) were then subjected to a repeated measures general mixed model (PROC MIXED) analysis with pen, calf breed, dam parity, week of measurement, and their first-order interactions fitted as fixed effects, while dam age was fitted as a random effect and all the initial body weight and conformation measurements fitted as covariates. We utilised an unstructured covariance structure because it provides the best fit for the analysis of repeated measurements based on a mixed model approach [18] compared to other covariance structures such as compound symmetric, spherical, and first order autoregressive [19]. The initial full statistical model used for the analysis was

Y = μ + P_i_ + B_j_ + D_k_ + W_l_ + (PB)_ij_ + (PD)_ik_ + (PW)_il_ + (BD)_jk_ + (BW)_jl_ + (DW)_kl_ + e_ijklm_

where Y = dependent variable, μ = overall mean, B_j_ = breed, D_k_ = dam parity, W_l_ = week of measurement, (PB)_ij_ + (PD)_ik_ + (PW)_il_ + (BD)_jk_ + (BW)_jl_ + (DW)_kl_ = first-order interactions, and e_ijklm_ = residual error.

All non-significant interactions (breed × dam parity, group pen × dam parity, dam parity × week of measurement) were removed from the final model. Linear, cubic, and quadratic orthogonal contrasts were fitted to test for significance as per Schad et al. [20]. Level of significance threshold was set at *p* < 0.05 and differences between means were established using Tukey’s probability pairwise comparison test.

## 3. Results

**Figure 1:**
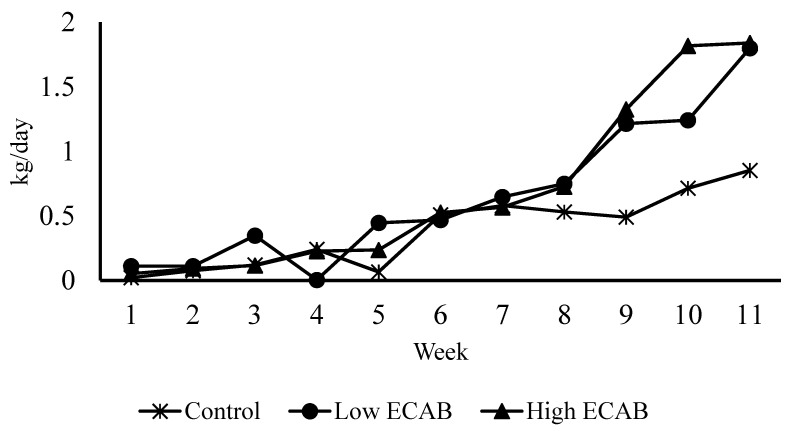
Weekly trends in average daily intake of calf starter feed supplemented with encapsulated calcium butyrate (ECAB).

**Figure 2:**
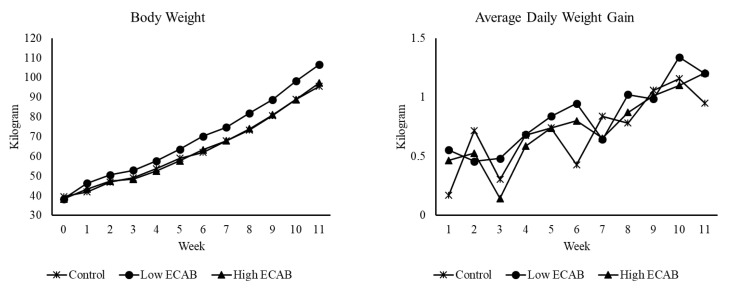
Effect of encapsulated calcium butyrate × week interaction on body weight (*p* < 0.05) and average daily gain (*p* < 0.001).

**Figure 3:**
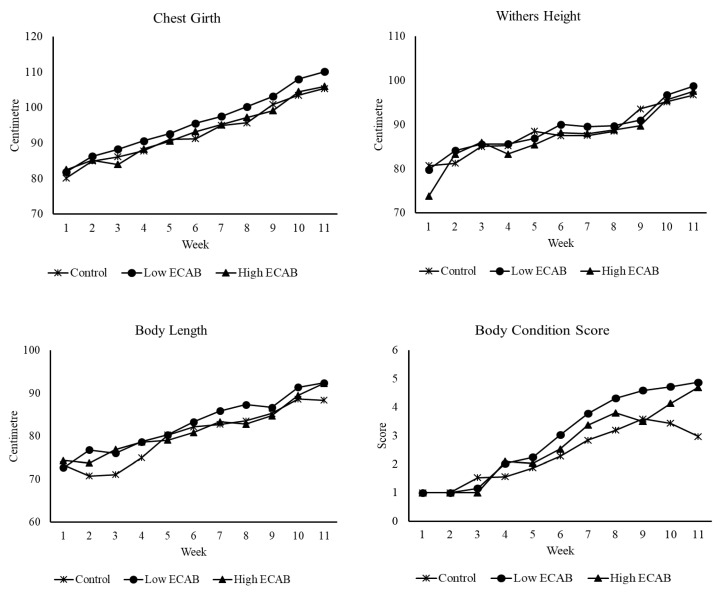
Effect of encapsulated calcium butyrate × week interaction on chest girth (*p* = 0.308), withers height (*p* < 0.001), body length (*p* < 0.001), and body condition score (*p* < 0.001).

The composition and nutrient analyses of the control and experimental diets as well as pastures grazed are depicted in Table 1 and Table 2. Since the post-weaning period did not involve any experimental feeding, the nutrient analysis of the pasture is only shown to provide an insight into the quality of the pasture the calves grazed during the post-weaning period. The results of the calculated average daily feed intake of the calf starter feed and growth parameters are presented in Table 3, Table 4 and Table 5, and Figure 1, Figure 2 and Figure 3.

### 3.1. Pre-Weaning Feed Intake

The nutrient compositions of the three experimental diets were isocaloric and isonitrogenous (same energy and crude protein contents) (Table 1) and the rye grass pasture was of high nutrient quality (Table 2). The feed intakes of calves fed low (4 kg/ton) and high (6 kg/ton) ECAB were numerically greater than for the control calves (Table 3). There was a weekly variation in daily feed intake among treatments, but a greater difference was observed from weeks 6 to 11 in Figure 1.

### 3.2. Pre-Weaning Growth and Body Conformation

The effect of ECAB supplementation on growth parameters is presented in Table 4. Overall, body weight (BWT) and average daily gain (ADG) were significantly (*p* < 0.05) greater for low ECAB calves compared to high ECAB or control calves. High ECAB and control calves were similar in BWT and ADG. For body conformation parameters, low ECAB calves had significantly greater (*p* < 0.05) chest girth (CG), longer (*p* < 0.05) body length (BL), and higher (*p* < 0.05) body condition score (BCS) than the high ECAB or control calves. Withers height (WH) was similar for control and low ECAB calves, and both were greater (*p* < 0.05) than high ECAB calves. High ECAB calves had similar CG, but longer (*p* < 0.05) BL and greater BCS when compared to control calves. There was a significant treatment by week interaction for BWT (*p* = 0.04) and ADG (*p* < 0.001) (Figure 2). Compared to other treatments, low ECAB calves were heavier from week one and maintained greater BWT throughout the 11 weeks of supplementary feeding. At the end of the feeding trial, calves fed Low ECAB weighed 106.6 ± 3.3 kg compared to control calves at 95.5 ± 3.1 kg and high ECAB calves at 97.2 ± 2.8 kg. There was also a significant treatment by week interaction for WH (*p* < 0.001), BL (*p <* 0.001) and BCS (*p* < 0.001), but not for CG (*p* = 0.3) as shown in Figure 3. There was also no significant treatment × breed interaction for BWT (*p* = 0.2), ADG (*p* = 0.5), CG (*p* = 0.2); WH (*p* = 0.1), BL (*p* = 0.1) and BCS (*p* = 0.6). Therefore, only the effect of breed is presented in Table 5. It shows that BWT was similar for Friesian and Friesian × Swedish Red crossbred calves and both were heavier (*p* < 0.05) than the Friesian × Jersey crossbred calves. Similar trends were observed for CG, WH, and BL. Although both Friesian and Friesian × Swedish Red calves had numerically higher ADG and BCS than Friesian × Jersey calves, the breed had no significant effect (*p* > 0.05).

### 3.3. Post-Weaning Growth

The results of post-weaning BWT and ADG of calves at 9 months (36 weeks or 252 days) of age are in Table 6. Body weight, total post-weaning weight gain, and post weaning ADG were similar for low and high ECAB calves, and both were greater (*p* < 0.05) than control calves. High ECAB calves had numerically higher total weight gain and ADG than low ECAB calves.

## 4. Discussion

Due to the fact that butyrate is the main short chain fatty acid that stimulates rumen epithelial growth, encourages an increase in rumen mitosis to apoptosis ratio, enables large rumen papillae growth, and increases the surface area for nutrient absorption [7,11], it becomes readily available in the digestive tract when consumed by neonatal calves. Few studies on supplementing calf starter rations with salts of butyric acid showed positive effects on the development of the rumen papillae, small intestinal villi, and the overall performance of young calves [12,13,14]. Earlier studies also demonstrated that butyric acid supplementation promoted the growth of epithelial cells of the gastrointestinal tract, improved digestion of feed, absorption of nutrients and immune function of the intestine [8,15]. Although the gastro-intestinal tract histology, physiology, or immune functions were not measured, we hypothesised that the improved growth performance observed in this study following supplementation of ECAB may have been due to the effect of supplemented butyrate on all or some of the physiological and histological effects previously reported in the literature [8,9,10,11,12,13,14,15]. When put together, the results of this study showed that the effect of supplementing neonatal calves with ECAB was better at 4 kg/ton of the calf starter compared to ECAB at 6 kg/t of calf starter feed. Supplementation at a higher dose of 6 kg/ton improved intake of the starter feed but did not significantly improve growth performance when compared with control calves. The reason for the lack of response at 6 kg/t of ECAB is not known but does indicate there may be no biological advantage of supplementing ECAB at 6 kg/t. The results for calves fed ECAB at 4 kg/ton are consistent with previous studies that showed positive effect by supplementing young calves with calcium butyrate [13,22,23] or sodium butyrate [14,24,25,26]. While some of these studies incorporated butyrate in both milk replacer and starter feeds [24], others incorporated butyrate into either the milk replacer [6,13,25] or starter feed [22]. In the present study, butyrate was supplemented in the starter feed while fresh milk was allocated at a fixed rate of 5.5 L per head per day through an auto feeder.

An increase in feed intake by calves supplemented with ECAB in this study is consistent with previous observations [6,14]. Supplementation of young calves with butyrate was shown to promote GIT development and function, which in turn led to higher starter feed intake in the later stage of calf growth [24]. There appears to be a positive correlation between solid feed intake in the early stages of life of a calf and solid feed intake in the later stage of rearing [24,27]. In this study, we observed an overall improvement in starter feed intake within the first five weeks of feeding, indicating that the effect of ECAB supplementation might have stimulated the necessary physiological and metabolic mechanisms that enhanced greater solid feed intake at a later stage of growth. In addition, dietary supplementation with butyrate might have stimulated accelerated passage through the GIT, and more efficient nutrient absorption and utilisation, leading to subsequent increase in starter feed intake that carried over to later developmental stage in life [6,14,24]. The greater intake observed with butyrate calves after 6–7 weeks of feeding coincides with when the rumen is expected to be fully physiologically functional and therefore, can support increased intake of solid feed. It is speculated from this study that the effect of butyrate supplementation could potentially have had a positive signaling effect right from the onset of feeding and this may have had a carry-over effect to a later stage of feeding. Delaying the onset of supplementation with butyrate probably does not have a similar effect on solid feed intake. Although the ADG for butyrate-supplemented calves in this study was higher than those reported in previous studies, the overall results on growth performance agree with those reported in previous studies [6,13,14,24,28,29]. These studies also found that the addition of butyrate to calf diets improved growth performance and increased feed conversion efficiency.

The positive effect on body conformation traits (chest girth, withers height, body length, and body condition) is consistent with the report of Ferreira and Bittar [30] who also showed that body conformation traits improved when calves were supplemented with sodium butyrate. It is expected that body weight gain is positively associated with body conformation traits. Contrary to this present study, Araujo et al. [31] and Wanat et al. [32] reported no significant advantage in the growth performance of dairy calves supplemented with butyrate over their control counterparts. This contrasting finding could not be explained clearly but may be related to the different type of butyrate used. For this study, encapsulated calcium butyrate was used, whereas sodium butyrate was used by Wanat et al. [32], while Araujo et al. [31] used both sodium butyrate and tributyrin. In the present study, butyrate supplemented calves had higher starter feed intake compared to the control calves, whereas Araujo et al. [31] and Wanat et al. [32] observed reduced feed intake and concluded that the lower starter feed intake may be related to possible keratinization of rumen papillae by butyrate [33], which can in turn negatively affect absorption of short-chain fatty acids from the rumen [34]. It is also possible that sodium butyrate may have affected palatability and therefore, intake of the milk replacer [31].

In the present study, supplementation of ECAB at 6 kg/ton did not result in any superior growth response compared to control calves. It appears that the dose and type of butyrate may interact with the diet and other factors to influence the effect of butyrate supplementation on calf growth performance. Our experience also suggests that at a higher dose greater than 6 kg/ton, supplementation with butyrate tends to affect palatability and drastically reduced total dry matter intake of the starter feed.

This study showed that growth performance and body conformation changed over time with ECAB supplementation, and low ECAB calves maintained higher BWT throughout the experimental period. Average daily gain and other body conformation parameters seem to vary over time, while low ECAB calves had better response overall. Calves supplemented with ECAB during the pre-weaning period continued to grow at a relatively higher rate compared to control calves, hence a large difference in final post weaning BWT.

In this study, the effect of ECAB was carried over through post-weaning and up to nine months of age. The fact that all calves were raised under the same management and pasture conditions suggests a true effect of supplementation with ECAB during the pre-weaning period. To the best of our current knowledge, no studies have reported any possible effect of pre-weaning supplementation of ECAB on post-weaning response. High ECAB calves had numerically higher post-weaning ADG when compared to low ECAB calves, resulting in both groups having similar BWT after nine months of age. It appears that the effect of pre-weaning supplementation with high ECAB was realized during the post-weaning period. Darvamanesh et al. [23] reported a significant effect of supplementing calcium butyrate on post-weaning ADG, but not on pre-weaning ADG. The post-weaning period in the study by Darvamanesh et al. [23] was from day 46 to day 67, whereas in the current study, the post-weaning period was between 90 days and up to 270 days. Therefore, the post-weaning period as reported by Darvamanesh et al. [23] is actually a corresponding period of pre-weaning for the current study. The post-weaning carry-over effect of ECAB may be due to pre-weaning effect on rumen and small intestine development, allowing for greater capacity for higher feed intake, digestion, and absorption during the post-weaning period. All these will result in improved feed intake and overall calf performance in terms of bodyweight and body conformation traits. The practical implication of this improved performance is on the lifetime performance of the calf. Accelerated growth can lead to early attainment of puberty and age at first calving, both of which have been shown to have a significant and positive impact on lifetime lactation of cows [3,34]. There is also the reproductive benefit when calves can achieve accelerated growth in the pre-weaning period so that they reach puberty and first calving at an early age [35,36].

## 5. Conclusions

Supplementing heifer calves with ECAB at a low dose of 4 kg/ton resulted in heavier calves, faster growth, wider chest girths, taller wither heights, and longer body lengths than unsupplemented calves and those on a high dose of 6 kg/ton of ECAB. Supplementation of calves with ECAB at 6 kg/ton did not improve any of the growth and body conformation parameters during the pre-weaning phase but improved post-weaning growth in a similar fashion to 4 kg/t ECAB when compared to the control diet. The outcomes of the current study taken together showed that pre-weaning supplementation of neonatal calves with ECAB in calf starter feed under a pasture-based dairy production system will likely support improved growth performance, both during pre-weaning and post-weaning periods, and this is most likely due to the effect of butyrate on GIT development and health. Supplementation with ECAB at a rate greater than 4 kg/ton may not have any additional growth benefits during the pre-weaning period but may confer a carry-over growth response during the post-weaning period. Practical implications of supplementing neonatal heifer calves with ECAB is the potential to wean calves early and reduce age at first calving, which has been shown to result in improved lifetime performance. Further studies are required to understand potential genetics and dietary interactions in supplementing neonatal calves with ECAB and the potential carry-over effect during the post-weaning period, and later during subsequent lactations.

## Figures and Tables

**Table 1:** Formulation and nutrient composition of the experimental feeds.

Item	Treatment ^1^		
	Control	Low ECAB	High ECAB
Ingredient (%)			
Wheat	39.9	39.9	39.9
Barley	26.0	26.0	26.0
Canola meal	25.0	25.0	25.0
Lupin	5.0	5.0	5.0
Molafos	1.0	1.0	1.0
Vegetable oil	0.5	0.5	0.5
Acid buf	0.5	0.5	0.5
Magnesium phosphate	0.3	0.3	0.3
Salt	0.5	0.5	0.5
Limestone	1.0	1.0	1.0
Mineral premix	0.3	0.3	0.3
High Five	0.1	0.1	0.1
Nutrient analysis (% DM basis)			
Dry matter	90.0	89.9	89.8
CP	22.7	23.1	20.8
ME, MJ/kg	12.9	12.8	13.1
NDF	15.3	16.3	14.0
Ether extract	3.7	3.9	3.3

^1^ Treatments: Low ECAB = 4 kg/ton encapsulated calcium butyrate; High ECAB = 6 kg/ton encapsulated calcium butyrate.

**Table 2:** Nutrient composition of ryegrass pasture at Willowdene Dairy Farm.

Nutrient	Composition (% DM)
DM	20.5
CP	13.3
ADF	26.7
NDF	43.8
EE	3.0
ASH	6.4
%TDN	62.3
DE (Mcal/kg)	2.7
ME (MJ/kg)	9.4

DM: Dry matter; NDF: Neutral detergent fibre; ADF: Acid detergent fibre; EE: Ether extract; CP: crude protein; %TDN: total digestible nutrients, calculated as (% of DM) = 82.38 − (0.7515 × ADF [% of DM]); ME: metabolisable energy, calculated by converting %TDN to digestible energy (DE [Mcal/kg] = %TDN × 0.01 × 4.4) which was converted as ME = (DE (Mcal/kg) × 0.82) × 4.185 as per Le et al. [21].

**Table 3:** Least square means (± SE) of starter feed intake of calves over eleven weeks (77 days) of feeding.

Item	Treatment ^1^			
	Control	Low ECAB	High ECAB	*p*-Value
Total feed intake/group, kg	47 1± 8.2	800 ± 8.7	844 ± 8.7	0.0445
Average daily feed intake/group, kg	6.1 ± 0.1	10.4 ± 0.3	11.0 ± 0.3	0.0315
Average daily feed intake/calf, g	382 ± 5.2	649 ± 6.4	688 ± 6.5	0.0475
Feed conversion efficiency	0.19 ± 0.01	0.37 ± 0.09	0.29 ± 0.06	0.0271

^1^ Treatments: Low ECAB = 4 kg/ton encapsulated calcium butyrate; High ECAB = 6 kg/ton encapsulated calcium butyrate.

**Table 4:** Effect of encapsulated calcium butyrate (ECAB) on body weight (BWT), average daily gain (ADG), chest girth (CG), withers height (WH), body length (BL), and body condition score (BCS) of calves over the entire 11 weeks (77days) of feeding ^1^.

Item	Treatment ^2^			*p*-Value
	Control	Low ECAB	High ECAB	
BWT, kg	65.4 ± 1.4 ^b^	72.1 ± 1.6 ^a^	65.7 ± 1.5 ^b^	0.0001
ADG, kg	0.71 ± 0.03 ^b^	0.83 ± 0.03 ^a^	0.74 ± 0.03 ^b^	0.0001
CG, cm	92.9 ± 0.7 ^b^	95.9 ± 0.7 ^a^	93.4 ± 0.7 ^b^	0.0001
WH, cm	88.2 ± 0.5 ^ab^	88.9 ± 0.5 ^a^	87.4 ± 0.7 ^b^	0.0001
BL, cm	80.1 ± 0.6 ^c^	82.9 ± 0.6 ^a^	81.5 ± 0.6 ^b^	0.0001
BCS	1.30 ± 0.08 ^c^	1.99 ± 0.12 ^a^	1.67 ± 0.10 ^b^	0.0001

^1^ Treatments: Low ECAB = 4 kg/ton encapsulated calcium butyrate; High ECAB = 6 kg/ton encapsulated calcium butyrate. ^2^ Means within row with different superscripts are different (*p* < 0.05). Values are least square means ± SE.

**Table 5:** Effect of breed on body weight (BWT), average daily gain (ADG), chest girth (CG), withers height (WH), body length (BL), and body condition score (BCS) over the entire 11 weeks (77days) of feeding ^1^.

Parameter	Breed			
	Friesian	Friesian × Jersey	Friesian × Swedish Red	*p*-Values
BWT, kg	65.9 ± 0.9 ^a^	58.8 ± 1.7 ^b^	67.9 ± 2.1 ^a^	0.0002
ADG, kg	0.70 ± 0.02	0.67 ± 0.04	0.79 ± 0.04	0.1410
CG, cm	93.4 ± 0.4 ^a^	90.1 ± 0.9 ^b^	93.5 ± 0.9 ^a^	0.0004
WH, cm	88.2 ± 0.3 ^a^	84.6 ± 0.5 ^b^	87.8 ± 0.7 ^a^	0.0001
BL, cm	80.7 ± 0.3 ^a^	78.9 ± 0.8 ^b^	82.1 ± 0.8 ^a^	0.0069
BCS	1.41 ± 0.1	1.46 ± 0.1	1.74 ± 0.2	0.3905

^1^ Means within row with different superscripts are different (*p* < 0.05). Values are least square means ± SE.

**Table 6:** Effect of pre-weaning supplementation with encapsulated calcium butyrate (ECAB) on post-weaning body weight (BWT), total weight gain (TWG), and average daily gain (ADG) ^1^ at 9 months of age ^1^.

Parameter	Encapsulated Calcium Butyrate Inclusion, kg/ton			*p*-Value	
	0	4	6	Treatment	Treatment × Breed
BWT, kg	199.9 ± 6.27 ^a^	227.9 ± 5.27 ^b^	227.2 ± 6.22 ^b^	0.0035	0.3872
TWG, kg	106 ± 4.64 ^a^	122.3 ± 3.9 ^b^	128.6 ± 4.61 ^b^	0.0047	0.7042
ADG, kg	0.697 ± 0.03 ^a^	0.805 ± 0.03 ^b^	0.846 ± 0.03 ^b^	0.0047	0.7042

^1^ Means within row with different superscripts are different (*p* < 0.05). Values are least square means ± SE.
